# Effects of Industrial Boiling on the Nutritional Profile of Common Octopus (*Octopus vulgaris*)

**DOI:** 10.3390/foods8090411

**Published:** 2019-09-12

**Authors:** Helena Oliveira, José António Muniz, Narcisa Maria Bandarra, Isabel Castanheira, Inês Ribeiro Coelho, Inês Delgado, Susana Gonçalves, Helena Maria Lourenço, Carla Motta, Maria Paula Duarte, Maria Leonor Nunes, Amparo Gonçalves

**Affiliations:** 1 IPMA, I.P., Portuguese Institute for the Sea and Atmosphere, Division of Aquaculture and Upgrading, Av. Alfredo Magalhães Ramalho 6, 1495-165 Lisboa, Portugal; joanmuniz@bol.com.br (J.A.M.); narcisa@ipma.pt (N.M.B.); sgoncalves@ipma.pt (S.G.); helena@ipma.pt (H.M.L.); amparo@ipma.pt (A.G.); 2 CIIMAR, Interdisciplinary Centre of Marine and Environmental Research, University of Porto, Terminal de Cruzeiros do Porto de Leixões, Av. General Norton de Matos S/N, 4450-208 Matosinhos, Portugal; nunes.leonor@gmail.com; 3 MEtRICs/DCTB, Faculty of Sciences and Technology, Universidade Nova de Lisboa, 2829-516 Caparica, Portugal; mpcd@fct.unl.pt; 4 INSA, I.P., National Institute of Health Dr. Ricardo Jorge, Food and Nutrition Department, Av. Padre Cruz, 1649-016 Lisboa, Portugal; isabel.castanheira@insa.min-saude.pt (I.C.); ines.coelho@insa.min-saude.pt (I.R.C.); ines.delgado@insa.min-saude.pt (I.D.); carla.motta@insa.min-saude.pt (C.M.)

**Keywords:** convenient seafood, healthy food, true retention, fatty acids, amino acids, elemental composition

## Abstract

Industrial cooking of common octopus (*Octopus vulgaris*) under well-established procedures is advantageous for current consumers, which demand healthy and convenient food. This work aimed to evaluate the effect of industrial water boiling, without the addition of salt, on the nutritional profile of common octopus. True retentions (TRs) were calculated for essential nutrients and toxic elements. After boiling, the moisture content decreased, resulting in a concentration of other constituents (protein, fat, fatty acids, majority of amino acids, phosphorus, zinc, and iodine). High TRs were obtained for some essential nutrients: 90.2% (eicosapentaenoic acid, EPA), 89.1% (docosahexaenoic acid, DHA), ≥74.6% (indispensable amino acids, IAA), and 86.8% (iodine). In both raw and boiled octopus, polyunsaturated fatty acids (252.2 and 425.1 mg/100 g), leucine (940.1 and 1613.4 mg/100 g), glutamate (1971.5 and 3257.1 mg/100 g), sodium (393.3 and 332.5 mg/100 g), and zinc (12.6 and 16.6 mg/kg) were, respectively, the most abundant fatty acids, IAA, dispensable amino acids, macro, and micro elements. Cadmium, lead, and mercury levels found in boiled octopus were 0.02, 0.10, and 0.08 mg/kg, respectively. The consumption of 150 g (usual portion) of boiled octopus is advisable because it contributes to significant daily intakes of EPA+DHA (>100%), selenium (75.6%), and iodine (12.4%), and 25% of the daily adequate intake of sodium for adults.

## 1. Introduction

The increasing demand towards seafood is mostly due to the increased awareness of their nutritional benefits [1]. Fishery products have a high protein content and are a rich source of indispensable amino acids (IAA), as well as other essential nutrients to human health, such as omega-3 polyunsaturated fatty acids (PUFA*n*-3) and minerals, in particular selenium and iodine, which cannot be acquired from other sources [1]. 

Cephalopods (octopuses, squids, and cuttlefishes) are among the most important groups of commercial marine species, representing approximately 4% of world total marine catch [2]. The most important octopus species, such as the common octopus, *Octopus vulgaris*, are commercially harvested worldwide. In southern European countries, such as Spain and Portugal, common octopus is one of the most important fishery resources in terms of market value [3,4]. The growing demand of this species commands the high prices throughout the distribution chain, and it is traditionally influenced by geographical and cultural reasons. Thus, octopus products are mostly consumed in southern Europe because consumers are familiar with such products, and also due to the increased awareness of its high versatility in terms of recipes and pleasant sensory properties. Live, fresh, and frozen are the most marketed products [5]. However, consumers report difficulties in cooking octopus at home, since very often its meat becomes tough, rubbery, and dry, compromising its acceptance. Additionally, the demand towards convenient cooked food is continuing to grow [1], which implies that food manufacturers and retailers are to largely play the role of cooking chefs. In such circumstances, the industrial preparation and cooking of common octopus under well-established procedures is of relevance for the seafood industry and a great advantage for consumers.

Currently, several culinary methods are used to cook fishery products, such as boiling, grilling, frying, steaming, baking, roasting, and microwaving. Boiling, which involves cooking in water at the boiling point (ca. 100 °C), is widely applied, because it is safe, simple [6], and appropriate for small and medium-scale food enterprises, since it requires low capital investment compared to emerging technologies such as ohmic heating and pulsed electric field with or without heating [7,8]. 

Although cooking makes seafood more palatable, digestible, and microbiologically safe [9], it may affect the food (including seafood) nutritional value [6,9,10,11,12,13]. In regard to cephalopods, some studies have been carried out on the composition and nutritional quality of raw edible part [3,14,15,16], but only one was found for boiled common octopus in a laboratory environment [17]. Published works focusing on the nutritional profile of common octopus boiled under industrial conditions were not found. On the other hand, it has been observed that processed foods contribute markedly to the sodium (Na) intake and the reduction of salt intake in population is declared among the five priority interventions by WHO [18]. Therefore, the role of the food industry and food services is critical in developing low-salt products, especially when the process is not affected by the absence of salt. In the case of octopus, cooking with salt addition is not essential, since this species is rich in Na [14]. Hence, this work aimed to (1) prepare a convenient product (frozen boiled) ready to be consumed cold (e.g., as a salad) or as an ingredient for cooked dishes; (2) evaluate the effect of water boiling under industrial conditions, without the addition of salt or additives, on the most relevant nutrients and some contaminants; and (3) provide the most useful nutritional data by using true retention (TR) calculations [10].

## 2. Materials and Methods

### 2.1. Raw Material and Water Boiling Procedure

The common octopus was purchased in an auction (Lisbon region) and immediately transported to the laboratory. The octopus was weighted (accuracy of 0.1 g), gutted, washed, allowed to drain, weighted again, frozen, and stored at −20 °C for three months. Water boiling of gutted octopus (previously thawed in a refrigerated chamber) was performed in a stainless steel gas tilting bratt pan (Berto’s model SBG9-15 I, Pádua, Italy, capacity: 110 L; superior calorific power: 19.11 kcal/h) over 45 min (without salt or other condiments) at the industrial cooking unit FASTER Produtos Alimentares Lda (Lousã, Portugal), which meets the Food IFS (International Featured Standard). Cooking time was previously optimized based on two main parameters: (i) Time to reach the internal temperature of 75 °C (microbiological safety criterion), using a penetration thermometer (Testo 105, Cabrils, Spain, accuracy of 0.5 °C) in the most anterior part of the octopus arms (corresponding to the thickest part); and (ii) time to achieve a suitable texture in the thickest portion of the arms, assessed by sensory evaluation. After boiling, the octopus was drained and cooled to room temperature. The final weight was registered to obtain the relevant cooking yield (CY = 100 × boiled weight/raw weight). Boiled octopus was quickly frozen and transported to the laboratory for analysis.

### 2.2. True Retention (TR)

The TR (%) for each nutrient and toxic element was calculated using the following formula (Murphy et al. [10]): TR = (content per g of cooked food)/(content per g of raw food) × CY. 

### 2.3. Analyses

Raw and boiled octopuses (i.e., thawed octopuses before and after boiling) were taken for several analyses, performed at least in duplicate. Results are given on a wet basis, as recommended by other authors [10,19]. According to Murphy et al. [10], inaccuracies could result from using retentions calculated on the dry basis. 

#### 2.3.1. Proximate Composition and Energy Value

Analyses were done according to the Association of Official Analytical Chemists (AOAC) methods [20]. The moisture content was quantified by oven drying at 105 ± 2 °C and the ash content by incineration of dry sample in a furnace at 500 ± 25 °C. The protein level was determined using an automatic nitrogen analyser LECO model FP-528 (LECO Corp., St. Joseph, USA). Nitrogen was released by combustion at 850 °C and detected by thermal conductivity. The total nitrogen was converted into protein using the factor of 6.25 for animal proteins [21]. Free fat was extracted with diethyl ether solvent in a Soxhlet apparatus (Behr Labor-Technik, Dusseldorf, Germany) during 7 h at approximately 40 °C. Fat content was determined by weighing the fat residue after drying in a 105 ± 1 °C air oven. The energy value was estimated using Food and Agriculture Organization (FAO) [22] factors.

#### 2.3.2. Fatty Acids

The fatty acid methyl esters (FAMEs) were prepared by acid-catalyzed transesterification according to the procedure of Lepage and Roy [23], modified by Cohen et al. [24]. An internal standard solution (C21:0) and acetyl chloride/methanol (1:19) were added. Samples were injected into a Bruker (model Scion 456, Livingston, UK) gas chromatograph equipped with an auto sampler. The separation of FAMEs was performed with helium as a carrier gas in a Stabilwax-MS polyethylene glycol capillary column (30 m length × 0.25 mm internal diameter, 0.25 μm thickness) programmed at 180 °C for 5 min, raised to 220 °C at 4 °C min^−1^, and maintained at 220 °C for 25 min. Detection of FAMEs was done on a flame ionization detector at 250 °C. The identification and quantification of FAMEs were accomplished through calibration curves using Sigma standards: Supelco PUFA No.1 (Marine Source, 99%-Ref. 47033) and PUFA No.3 (Menhaden oil 99%-Ref. 47085-U). 

The atherogenic index and thrombogenic index (AI and TI, respectively) were determined for evaluation of the predisposition for incidence of coronary heart disease according to Ulbricht and Southgate [25]: AI = (C12:0 + (4 × C14:0) + C16:0)/(∑MUFA + ∑PUFA*n*-3 + ∑PUFA*n*-6); TI = (14:0 + 16:0 + 18:0)/(0.5 × ∑MUFA) + (0.5 × ∑PUFA*n*-6) + (3 × ∑PUFA*n*-3 + (PUFA*n*-3/PUFA*n*-6)), where MUFA = monounsaturated fatty acids.

The hypocholesterolemic to hypercholesterolemic ratio (h/H) was also calculated according to Santos-Silva et al. [26]: h/H = (C18:1*n*-9 + C18:2*n*-6 + C20:4*n*-6 + C18:3*n*-3 + C20:5*n*-3 + C22:5*n*-3 + C22:6*n*-3)/(C14:0 + C16:0).

#### 2.3.3. Total Amino Acids (AA)

The protein hydrolysis of freeze-dried samples was undertaken on a microwave digestion system Milestone ETHOS 1 Series (Sorisole, Italy) under anaerobic conditions, using hydrochloric acid (6 N, containing 0.5% phenol) and an internal standard (d-Norvaline). The neutralized hydrolysates were submitted to pre-column derivatization using a buffer and a reconstituted Waters^®^ AccQ Fluor Reagent Kit (containing 6-aminoquinolyl-*N*-hydroxysuccinimidyl carbamate) at 55 °C. The derivatised AA separation was carried out on an Acquity UPLC system from Waters^®^ (Milford, MA,USA), equipped with a BEH C18 column (100 × 2.1 mm i.d., 1.7 m) using a mobile phase through a gradient. The detection was done on a photodiode array detector at 260 nm [27]. The identification and quantification were done by comparison with a standard AA mixture (AA Standard Hydrolysate and d-Norvaline, Waters^®^ mixed with taurine and tryptophan standards, Sigma) through calibration curves using the empower software from Waters^®^. The acid hydrolysis conditions (under rigid exclusion of oxygen) allow a considerable recovery of sulphur containing AA (cystine, cysteine, and methionine), and also tryptophan, particularly due to the very low levels of carbohydrate in the fishery products [28]. 

#### 2.3.4. Macro, Micro, and Toxic Elements

Magnesium (Mg), potassium (K), sodium (Na), copper (Cu), and zinc (Zn) were determined by flame atomic absorption spectrophotometry (Spectr AA 55B spectrophotometer, Varian, Palo Alto, CA, USA) with a background deuterium correction, based on the method described by Jorhem [29]. The concentrations were calculated using linear calibration obtained from absorbance measurements of, at least, five different concentrations of standard solutions: Mg(NO_3_)_2_, KNO_3_, NaNO_3_, Cu(NO_3_)_2_, and Zn(NO_3_)_2_ (dissolved in 0.5 M HNO3).

Phosphorus (P) was determined by molecular absorption spectrophotometry (UV/Vis UV2 spectrophotometer, UNICAM, Algés, Portugal) according to ISO Standard 13730 [30]. Samples were dry-ashed at 500 ± 25 °C, followed by acid digestion and colorimetric measurement of a yellow compound resulting from the reaction between P and an ammonium vanadate and ammonium molybdate mixture at 430 nm. 

Selenium (Se) was determined according to the EN 15763:2009 [31]. Samples were digested with 2% (*v*/*v*) nitric acid solution in a microwave digestion system (Milestone ETHOS 1 Series, Sorisole, Italy)). The iodine (I) content was quantified according to the EN 15111:2007 [32]. The extraction was performed by a graphite block system with Tetramethylammonium hydroxide (TMAH) solution 25% (*v*/*v*) (Fluka). Se and I were determined by an inductively coupled plasma mass spectrometer (ICP-MS, Thermo X series II, Leicestershire, UK). Working standard solutions of Se and I were prepared from single elements high purity ICP stock standards (SCP Science and Inorganic Ventures, respectively). Internal standard solutions of germanium, indium, and yttrium (Inorganic Ventures) and of rhodium (Inorganic Ventures) and tellurium (Merck) were used, respectively, for Se and I. Quantification was done by comparison with calibration curves using the software XseriesPlasmaLab 2.5 (Leicestershire, UK).

Cadmium (Cd) and lead (Pb) were determined by graphite furnace atomic absorption spectrometry according to the EN 14084 [33]. A spectrophotometer apparatus (Spectr 220Z, Varian, Palo Alto, CA, USA) with a Zeeman correction (*λ* = 228.8 and 283.3 nm for Cd and Pb, respectively) was used. The concentrations were measured through linear calibration obtained from absorbance measurements of, at least, five different concentrations of standard solutions: Cd(NO_3_)_2_ and Pb(NO_3_)_2_ (1 g L^−1^ dissolved in 0.5 M HNO_3_).

Total mercury (Hg) was quantified on a direct mercury analyser spectrophotometer (AMA 254, LECO Corp., St. Joseph, MI, USA) according to the methodology described in EPA [34]. The samples were thermally decomposed followed by oxidation and amalgamation. The Hg absorption was read at 253.7 nm wavelength using a calibration curve range of 0–36 ng.

#### 2.3.5. Analytical Quality Assurance

Analytical quality was assessed (Table 1 and Table 2) according to the procedures described in Magnusson and Örnemark [35]. The reagents used were of analytical grade.

### 2.4. Nutritional Contribution (NC)

The NC of boiled common octopus was calculated based on eicosapentaenoic acid (EPA) + docosahexaenoic acid (DHA), macro and micro elements, considering a portion of 0.150 kg and the dietary reference values (DRVs) recommended by the European Food Safety Authority [36,37,38,39,40,41,42,43,44], according to the following formula: NC (%) = 100 × (C × M)/AI*, where C = mean concentration of the nutrient (EPA+DHA or mineral) in mg/kg; M = typical meal portion (0.150 kg); AI = adequate intake (mg/day); and *PRI (Population Reference Intake (mg/day)) used in the case of zinc.

The DRVs for AA remain questionable since, in general, AA are provided in the form of protein [45]. Thus, the above formula was not applied to IAA and the protein quality was estimated based on the AA scores according to WHO [46]: AA score (%) = 100 × IAA (mg/g protein)/IAA in FAO [47] reference pattern (mg/g protein).

### 2.5. Statistical Treatment

Statistical analysis was performed with the program STATISTICA version 7.0 (StatSoft Inc., Tulsa, OK, USA). The influence of the water boiling on the chemical parameters was tested by the Student *t*-test. Statistical significance was considered at *p* < 0.05 for all analyses [48].

## 3. Results and Discussion

### 3.1. Cooking Weight Yield

Water boiling under industrial conditions resulted in a CY of 52.8%, a value comparable to those reported by Mendes et al. [4] and Rosas-Romero et al. [49] for steam-cooked common octopus and jumbo squid, respectively. The usual low cooking yields result from the damaging and solubilization of the high proportion of connective tissue belonging to the musculature of octopus and the disconnection and dehydration of the muscle fibrils [49,50].

### 3.2. Proximate Composition and Energy Value

Cooking techniques that require heat can affect the nutritional composition of seafood depending on the intrinsic composition, temperature, and time the product is exposed and the method used. The moisture content found in raw octopus was significantly higher than in cooked flesh, resulting in a TR of 47.8% (Table 3). The water loss may be attributed to the evaporation, dehydration of the muscle fibrils, and probably to some heat-induced protein denaturation during boiling, which causes less water to be entrapped within the protein structures [51]. Concerning proteins, it is known that they are not easily lost during cooking since they undergo physical changes, mainly denaturation and coagulation [6,50]. In the case of octopus boiling, some solubilization and, hence, some losses took place, nevertheless a high retention (78.8%) was observed. The total fat was also highly retained (89.5%) in cooked octopus because it is mainly composed of phospholipids, which are structural compounds of which the majority are found in cell membranes (as usual in lean species), whose release by heat is more difficult than the triacylglycerols. TR found for protein and fat is in accordance with the values reported by Bógnar [11] for boiled molluscs-based dishes such as squid (edible part). Similar effects of boiling under industrial conditions on proximate composition were observed in a laboratorial environment with the same species caught in the Portuguese coast and other fish species [9,17,51]. As a consequence of the increase of the protein and fat contents, the energy value increased to 120 kcal/100 g in boiled octopus.

### 3.3. Fatty Acids

The PUFA were the predominant, followed by saturated fatty acids (SFA) and MUFA (around 59, 30, and 11% of the total fatty acids, respectively) in raw and boiled samples (Table 4). Among PUFA, EPA+DHA (omega 3 fatty acids), which are vital for human health, represented a considerable percentage of the total fatty acids (42%). The highest levels of SFA and MUFA were reported for C16:0 and C20:1n-9 (approximately 59% and 30% of the total of SFA and MUFA, respectively). Boiling resulted in high TRs for all fatty acids, which ranged from 82.6 to 100.9%. Most of the retention values found for fatty acids were similar to those reported for boiled catfish and baked European sea bass in aluminium foil (i.e., moist heat) [52,53].

The high quality of the lipids is reflected by the values obtained for the PUFA*n*-3/PUFA*n*-6 ratio (≈5), revealing the importance of this species as a significant dietary source of PUFA*n*-3 and its beneficial role in human health. Bandarra et al. [17] and Chakraborty et al. [16] also reported PUFAn-3/PUFAn-6 ratios higher than 5 for octopus species.

Water boiling did not affect the AI and TI or the h/H ratio. The AI and TI results were lower than those reported by Chakraborty et al. [16] for other raw octopus species, and Costa et al. [51] for raw and boiled meagre. The low AI and TI values obtained suggest a high cardio-protective effect, since these indices represent the balance between the promotion and protection of coronary heart diseases [25]. The h/H values observed were within the range of those mentioned by Chakraborty et al. [16] for other raw cephalopods species and also indicate that common octopus is nutritionally beneficial for human health [54]. 

### 3.4. Amino Acids

Like in most seafood products, glutamate and aspartate were the dominant AA [52] (the sum of both corresponded to approximately 40% of the total dispensable amino acids), while cystine+cysteine had the lowest amount in both raw and boiled octopus (Table 5). Among IAA, leucine was the most abundant, followed by lysine (both corresponding to approximately 40% of the IAA), while tryptophan had the lowest content (close to 3% of the IAA). These levels were similar to those mentioned by Bandarra et al. [17]. The IAA represented 31.3% and 35.5% of the total AA in raw and boiled octopus, respectively, percentages lower than those reported by Chakraborty et al. [16]. However, these authors did not present the levels of taurine, that are quite relevant in raw and boiled octopus (Table 5). Such levels contribute to the interest of the octopus inclusion into a balanced diet, since this AA has several biological effects in different human organs or systems (e.g., cardiovascular and nervous systems) [55].

High retentions were found for AA, ranging from 65.7 to 98.9%, with the exception of taurine (43.4%), as expected since it is a free AA. The TRs observed for AA are in agreement with the high TRs found for protein that undergo mainly physical changes instead of losses during cooking [6,50]. Consequently, the balanced AA composition is maintained after boiling. The TRs obtained for IAA ranged from 74.6% (tryptophan) to 98.9% (lysine). Bógnar [11] also mentioned a high retention for methionine and lysine (95%, estimated values) for boiled molluscs-based dishes.

### 3.5. Macro, Micro, and Toxic Elements

The most abundant macroelement was Na, followed by K, P, and finally Mg in raw and boiled common octopus (Table 6). The Na content found imparts a suitable salty taste, as assessed by a sensory panel (data not shown). Thus, the use of salt to cook octopus is not necessary. Within the microelements, Zn was the dominant, followed by Cu. Identical patterns in the abundance of these elements were found for the same species by Bandarra et al. [17] and Lourenço et al. [14]. The levels of Na, K, P, Mg, Zn, and Cu in raw samples were within the range of those reported by Lourenço et al. [14] and Rjeibi et al. [15]. Regarding Se, Lourenço et al. [14] found a lower value (≈0.1 mg/kg) for the same species than that observed in this study (≈0.4 mg/kg). Similar values of this non-metal were mentioned by Costa et al. [51] for raw and boiled meagre (≈0.3 mg/kg). The I content was 0.08 mg/kg and 0.12 mg/kg in raw and boiled octopus, respectively. A significant variation between raw and boiled octopus was observed only for P, Zn, and I. An opposite result (lower contents in boiled products) was reported for Zn and P levels in some fish species [12,13]. Regarding I, Erkan [56] reported both losses and concentration of the contents after steam-cooking in different fish species. 

The TR ranged from 44.6% (Na) to 66.8% (P) and from 53.5% (Se) to 86.8% (I) for macro and microelements, respectively (Table 6). The low retentions observed could be ascribed to the leaching out of minerals to the boiling water, as suggested by Murphy et al. [10]. Other authors found higher retentions (≥75%) for the same macroelements, Zn and Cu, in boiled molluscs-based dishes and boiled catfish [11,52]. However, the values found by Bógnar [11] are estimated retention values.

Concerning toxic elements, the Cd, Pb, and Hg levels (Table 6) found in all samples were below the established European limits (1.0, 0.3, and 0.5 mg/kg, respectively) [57,58]. Hence, the high TR observed for Hg is not a problem. These low values are in agreement with those obtained by Lourenço et al. [14] and Raimundo et al. [59], which indicate that the *O. vulgaris* from the Portuguese coast does not constitute a public health concern.

### 3.6. Nutritional Contribution

#### 3.6.1. Protein Quality

AA are mainly obtained from proteins in the diet, and the dietary protein quality is related to its content of IAA. High quality proteins are easier to digest and have IAA levels similar to human requirements [46]. The results found indicate that common octopus is a species that has high quality protein and boiling does not induce losses in this quality, as can be confirmed by the AA scores above 100% (Table 7). An exception was found for valine (84.3%), but also in this case, its level almost met the recommended requirement by FAO for adults (>18).

#### 3.6.2. Mineral Elements and Fatty Acids (EPA + DHA)

The consumption of 150 g (usual portion) of boiled common octopus contributes, in terms of macroelements, 10.4% (K) to 56.0% (P) (Table 8) of the daily adequate intake for adults. In the particular case of Na, the NC is 24.9%, corresponding to 1.3 g of salt, which is far below the limit value recommended by EFSA (≈5 g of salt) [44]. Such contribution imparts an adequate salty taste, as assessed by a sensory panel (data not shown). Hence, the use of salt to cook octopus is not necessary, as already mentioned, and should be avoided, since it is well known that the high salt consumption contributes to raise blood pressure and increases the risk heart disease and stroke. Reducing salt intake to recommended levels is a priority and could prevent 2.5 million deaths every year [18,44]. In terms of microelements, the NC ranges from 25.5 to 33.2% for Cu and Zn, and it is 12.4% and 75.6% for I and Se, respectively. Thus, industrially boiled common octopus is a particularly good source of Se, which is essential for combating oxidative stress-induced diseases [16]. Despite the lower NC of iodine, the consumption of boiled octopus (150 g) contributes to reduce iodine deficiency, the most common cause of preventable mental impairment worldwide. It is known that nearly one-third of the global population still has inadequate dietary iodine intakes [36]. Additionally, a portion of 150 g fulfils the daily adequate intake of EPA+DHA (>100%) suggested by EFSA [42], based on considerations of cardiovascular health for adults. Hence, boiled common octopus should be consumed by individuals with cardiovascular ailments and can be considered a nutritious food product.

## 4. Conclusions

Industrial water boiling of common octopus causes a reduction in the moisture content, which results in a concentration of protein, fat, all fatty acids, the majority of AAs, and some elements (P, Zn, and I). High retentions were found for essential nutrients, namely EPA, DHA, IAA, and I. PUFA, leucine, Na, and Zn were the most abundant fatty acids, IAA, macro- and microelements, respectively. Furthermore, industrially boiled common octopus is an important source of high quality protein (majority of IAA scores >100%). The consumption of 150 g (usual portion) contributes to important daily intakes of beneficial nutrients for human health such as EPA+DHA (NC > 100%), Se (NC = 75.6%), and I (NC = 12.4%) in adults. In such portion, the Na content corresponds to less than half of the European limit recommended per day (NC = 25%) and confers an adequate salty taste. In terms of the toxic elements (Cd, Pb, and Hg), the Portuguese common octopus does not constitute any concern, since their contents were far below the established European limits. Results show that industrially boiled common octopus prepared without salt addition is a nutritious food product, which can be consumed by individuals with cardiovascular ailments (e.g., its consumption promotes a high cardio-protective effect). Thus, boiled common octopus can be prepared by the industry and supplied as a convenient product (frozen boiled) ready to be consumed cold (e.g., as a salad) or as an ingredient for cooked dishes. Additionally, true retention values presented are useful and meaningful nutritional data for key stakeholders such as seafood industry, nutritionists, dietetic associations, and consumers. Energy- and time-saving emerging technologies that are being used in the seafood industry, such as ohmic heating or pulsed electric field, should be considered in the coming studies related to the industrial cooking of common octopus.

## Figures and Tables

**Table 1:** Quality assurance of the proximate composition, fatty acids and macro, micro, and toxic elements analyses (*n* = 4).

Elements	Technique	DL	Proficiency Test or CRM	Certified ^1^	Present work ^2^
Proximate composition (g/100 g)					
Moisture	Drying	n.a.	FAPAS Test 01120	66.64 ± 0.47	66.44 ± 0.12
Ash	Incineration	0.16		2.12 ± 0.08	2.13 ± 0.01
Nitrogen	Combustion	0.0004		2.17 ± 0.04	2.20 ± 0.00
Fat	Soxhlet extraction	0.1		15.53 ± 0.47	15.88 ± 0.40
Fatty acids (g/100 g)					
14:0 16:0 16:1 18:0 18:1 18:2n-6 18:3n-3 20:5n-3 22:5n-3 22:6n-3	GC–FID	4 × 10^−6^–10 × 10^−6^	SRM-1946	0.316 ± 0.009 1.22 ± 0.04 0.816 ± 0.026 0.263 ± 0.011 2.64 ± 0.08 0.348 ± 0.023 0.221 ± 0.025 0.296 ± 0.019 0.335 ± 0.026 0.92 ± 0.10	0.238 ± 0.006 1.14 ± 0.04 0.735 ± 0.023 0.260 ± 0.005 2.39 ± 0.06 0.338 ± 0.009 0.200 ± 0.005 0.304 ± 0.009 0.323 ± 0.024 0.93 ± 0.10
Macroelements (mg/kg)					
Magnesium	FAAS	0.02	Dorm-4	910 ± 80	835 ± 12
Potassium	FAAS	0.01	Dorm-4	15500 ± 1000	14500 ± 495
Sodium	FAAS	0.09	FAPAS Test 01120	0.60 ± 0.03	0.55 ± 0.02
Phosphorus	MAS	0.01	n.d.	n.d.	n.d.
Microelements (mg/kg)					
Copper	FAAS	0.02	Dorm-4	15.7 ± 0.5	15.4 ± 0.7
Zinc	FAAS	0.06	Dorm-4	51.6 ± 2.8	48.4 ± 1.0
Selenium	ICP-MS	6.4 × 10^−3^	ERM^®^-BB422	1.33 ± 0.13	1.20 ± 0.02
Iodine	ICP-MS	7.8 × 10^−3^	n.d.	n.d	Recovery = 111%
Toxic elements (mg/kg)					
Cadmium	GFAAS	0.002	Dorm-4	0.299 ± 0.018	0.298 ± 0.011
Lead	GFAAS	0.02	Dorm-4	0.404 ± 0.062	0.412 ± 0.037
Mercury	AAS	0.004	Dorm-4	0.412 ± 0.036	0.407 ± 0.049

^1^ Values are presented as average ± uncertainty or ± standard deviation for fatty acids; ^2^ Values are presented as average ± standard deviation. Abbreviations: DL, detection limit; CRM, certified reference material; n.a., not applicable; n.d., not determined; GC–FID, Gas Chromatography–Flame Ionization Detection; FAAS, Flame Atomic Absorption Spectrometry; MAS, Molecular absorption Spectrophotometry; ICP-MS, Inductively Coupled Plasma Mass Spectrometry; GFAAS, Graphite Furnace Atomic Absorption Spectrometry; AAS, Atomic Absorption Spectrometry; FAPAS Test 01120, Nutritional Components in Canned Meat, January–March 2018 (Fera Science Ltd., York, UK); SRM-1946, Standard Reference Material from Lake Superior Fish Tissue (National Research Council of Canada, Canada); Dorm-4, fish protein certified reference material for trace metals (National Research Council of Canada, Canada); ERM^®^-BB422, European certified reference material (fish muscle) for selenium.

**Table 2:** Quality assurance of amino acids analysis (acid hydrolysis under anaerobic conditions followed by liquid chromatography (UPLC)) using a certified reference material NIST 3244 * (*n* = 3).

Amino Acid (g/100 g)	Detection Limit	Certified Value Average ± U ^1^	Analyzed Values Average ± SD ^2^
Alanine	1.13 × 10^−3^	2.12 ± 0.96	2.02 ± 0.01
Arginine	2.49 × 10^−3^	2.26 ± 0.52	2.15 ± 0.19
Aspartate	2.68 × 10^−3^	5.29 ± 0.28	5.00 ± 0.18
Cysteine	3.18 × 10^−3^	0.48 ± 0.14	0.32 ± 0.07
Glutamate	2.82 × 10^−3^	14.3 ± 2.10	14.64 ± 0.28
Glycine	1.01 × 10^−3^	1.23 ± 0.13	1.32 ± 0.08
Histidine	2.78 × 10^−3^	1.73 ± 0.17	1.52 ± 0.07
Isoleucine	0.85 × 10^−3^	3.00 ± 0.61	2.56 ± 0.04
Leucine	1.23 × 10^−3^	6.16 ± 0.88	5.78 ± 0.05
Lysine	3.79 × 10^−3^	4.78 ± 0.77	4.07 ± 0.44
Methionine	2.02 × 10^−3^	1.71 ± 0.28	1.66 ± 0.03
Phenylalanine	3.08 × 10^−3^	3.48 ± 0.50	3.67 ± 0.28
Proline	0.82 × 10^−3^	6.64 ± 0.73	6.36 ± 0.19
Serine	1.00 × 10^−3^	3.80 ± 0.35	3.16 ± 0.06
Threonine	1.11 × 10^−3^	2.76 ± 0.54	2.28 ± 0.03
Tryptophan	2.53 × 10^−3^	0.84 ± 0.29	0.51 ± 0.01
Tyrosine	3.94 × 10^−3^	3.16 ± 0.71	3.36 ± 0.26
Valine	0.73 × 10^−3^	3.67 ± 0.98	3.28 ± 0.04

* NIST 3244, Ephedra-containing protein powder. National Institutes of Standards and Technology, Gaithersberg, MD, USA. ^1^ Uncertainty; ^2^ Standard Deviation.

**Table 3:** Proximate composition, energy value, and true retention (TR) of common octopus.

Proximate Composition (g/100 g)	Raw ^1^	Boiled ^1^	TR (%)
Moisture	80.3 ± 1.0 ^a^	72.8 ± 1.1 ^b^	47.8
Ash	1.72 ± 0.07	1.60 ± 0.04	49.2
Protein	16.9 ± 1.1 ^a^	25.2 ± 0.9 ^b^	78.8
Fat	0.43 ± 0.03 ^a^	0.72 ± 0.03 ^b^	89.5
Energy value (kcal/100 g) ^2^	81 ± 5 ^a^	120 ± 4 ^b^	

^1^ Values are means ± standard deviation (*n* = 3). Different superscript letters within a row represent statistical differences (*p* < 0.05). ^2^ The glycogen value used (1.3%) for the calculation of the energy value was obtained from Rosa et al. [3].

**Table 4:** Fatty acids composition (most abundant) and true retention (TR) of common octopus.

Fatty Acids (mg/100 g)		Raw ^1^	Boiled ^1^	TR (%)
Miristic	14:0	3.40 ± 0.92 ^a^	5.85 ± 1.08 ^b^	90.9
Palmitic	16:0	72.91 ± 5.08 ^a^	128.77 ± 8.16 ^b^	93.2
Stearic	18:0	35.26 ± 5.81 ^a^	55.18 ± 4.90 ^b^	82.6
Total saturated	ΣSFA	125.74 ± 12.70 ^a^	213.20 ± 9.23 ^b^	89.5
Vaccenic	18:1*n*-7	7.28 ± 0.52 ^a^	13.92 ± 1.36 ^b^	100.9
Oleic	18:1*n*-9	9.58 ± 2.24 ^a^	17.50 ± 3.33 ^b^	96.4
11-Eicosenoic	20:1*n*-9	14.99 ± 2.13 ^a^	24.00 ± 1.23 ^b^	84.5
Total monounsaturated	ΣMUFA	47.68 ± 4.83 ^a^	83.24 ± 0.98 ^b^	92.1
Linoleic	18:2*n*-6	1.65 ± 0.20 ^a^	2.89 ± 0.32 ^b^	92.2
Arachidonic	20:4*n*-6	29.00 ± 3.78 ^a^	51.20 ± 3.36 ^b^	93.2
Eicosapentaenoic (EPA)	20:5*n*-3	77.20 ± 3.97 ^a^	131.89 ± 11.46 ^b^	90.2
Docosapentaenoic	22:5*n*-3	6.97 ± 0.92 ^a^	11.53 ± 0.63 ^b^	87.3
Docosahexaenoic (DHA)	22:6*n*-3	100.43 ± 4.96 ^a^	169.50 ± 5.07 ^b^	89.1
Total polyunsaturated	ΣPUFA	252.18 ± 17.59 ^a^	425.05 ± 22.15 ^b^	89.0
	EPA+DHA	177.63 ± 8.40 ^a^	301.39 ± 15.74 ^b^	89.5
Total omega 3	ΣPUFA*n*-3	210.10 ± 11.31 ^a^	354.02 ± 20.98 ^b^	88.9
Total omega 6	ΣPUFA*n*-6	40.34 ± 5.79 ^a^	68.63 ± 4.17 ^b^	89.8
	PUFA*n*-3/PUFA*n*-6	5.25 ± 0.46	5.17 ± 0.37	-----
Thrombogenic index	TI	0.16 ± 0.01	0.17 ± 0.00	-----
Atherogenic index	AI	0.29 ± 0.01	0.30 ± 0.02	-----
Hypocholesterolemic to hypercholesterolemic ratio	h/H	2.95 ± 0.08	2.86 ± 0.10	-----

^1^ Values are means ± standard deviation (*n* = 3). Different superscript letters within a row represent statistical differences (*p* < 0.05).

**Table 5:** Amino acids profile and true retention (TR) of common octopus.

Amino Acid	Raw ^1^	Boiled ^1^	TR (%)
	mg/100 g		
Indispensable (IAA) ^2^			
Histidine	275.5 ± 15.1 ^a^	441.2 ± 41.8 ^b^	84.5
Isoleucine	479.3 ± 25.4 ^a^	897.3 ± 115.8 ^b^	98.8
Leucine	940.1 ± 60.9 ^a^	1613.4 ± 126.9 ^b^	90.6
Lysine	660.5 ± 5.7 ^a^	1238.1 ± 100.3 ^b^	98.9
Methionine	347.8 ± 18.6 ^a^	568.5 ± 42.9 ^b^	86.3
Phenylalanine	544.8 ± 38.8 ^a^	861.1 ± 72.1 ^b^	83.4
Threonine	495.4 ± 26.0 ^a^	798.3 ± 41.5 ^b^	85.0
Tryptophan	127.1 ± 8.6 ^a^	179.7 ± 9.7 ^b^	74.6
Valine	454.7 ± 25.2 ^a^	828.2 ± 96.6 ^b^	96.1
∑IAA	4104.9 ± 584.3 ^a^	7425.8 ± 632.9 ^b^	
Dispensable (DAA) ^2^			
Alanine	729.6 ± 53.7 ^a^	1116.7 ± 81.0 ^b^	80.8
Arginine *	1049.6 ± 26.3 ^a^	1594.3 ± 154.9 ^b^	80.2
Aspartate	1325.0 ± 66.5 ^a^	2239.7 ± 136.6 ^b^	89.2
Cystine + Cysteine *	72.8 ± 11.6 ^a^	111.0 ± 5.3 ^b^	79.7
Glutamate	1971.5 ± 84.7 ^a^	3257.1 ± 206.3 ^b^	87.2
Glycine *	1179.5 ± 302.1	1467.6 ± 163.5	65.7
Proline *	698.7 ± 114.9 ^a^	957.8 ± 54.8 ^b^	72.3
Serine	615.6 ± 13.5 ^a^	904.4 ± 41.3 ^b^	77.5
Taurine *	1225.0 ± 142.9	1007.6 ± 124.8	43.4
Tyrosine *	539.5 ± 40.3 ^a^	841.7 ± 70.2 ^b^	82.3
∑DAA	9407.0 ± 371.6 ^a^	13496.9 ± 973.8 ^b^	
∑AA	13511.9 ± 714.6 ^a^	20922.7 ± 1606.7 ^b^	
∑IAA/∑AA (%)	30.3 ± 3.2 ^a^	35.5 ± 0.3 ^b^	

^1^ Values are means ± standard deviation (*n* = 3). Different superscript letters within a row represent statistical differences (*p* < 0.05). ^2^ According to WHO [46]. * Conditionally indispensable (amino acids that can become indispensable under specific physiological or pathological conditions) [46]. ∑AA, total amino acids.

**Table 6:** Concentration of macro, micro, and toxic elements and true retention (TR) of common octopus.

	Raw ^1^	Boiled ^1^	TR (%)
Macroelements (mg/100 g)			
Mg	57.4 ± 2.5	56.1 ± 2.4	51.6
P	162.2 ± 6.5 ^a^	205.2 ± 13.3 ^b^	66.8
K	262.2 ± 24.1	241.7 ± 27.9	48.6
Na	393.3 ± 41.6	332.5 ± 24.3	44.6
Microelements (mg/kg)			
I	0.08 ± 0.01 ^a^	0.12 ± 0.01 ^b^	86.8
Se	0.35 ± 0.06	0.35 ± 0.01	53.5
Cu	1.8 ± 0.4	2.7 ± 0.6	80.5
Zn	12.6 ± 0.6 ^a^	16.6 ± 0.8 ^b^	69.8
Toxic elements (mg/kg)			
Hg	0.04 ± 0.01 ^a^	0.08 ± 0.02 ^b^	95.7
Cd	0.03 ± 0.02	0.02 ± 0.00	42.5
Pb	<0.06 *	0.10 ± 0.03	----

^1^ Values are means ± standard deviation (*n* = 3). Different superscript letters within a row represent statistical differences (*p* < 0.05). * Limit of quantification.

**Table 7:** Indispensable amino acid concentration and amino acid (AA) scores of boiled common octopus and Food and Agriculture Organization (FAO) reference standard concentration for IAA.

Amino Acid	Concentration (mg/g Protein)		AA Score (%) ^1^
	FAO Standard *	Boiled Octopus ^1^	
Histidine	15	17.5 ± 1.9	116.8 ± 12.4
Isoleucine	30	35.6 ± 4.6	118.7 ± 15.2
Leucine	59	64.1 ± 5.7	108.6 ± 9.6
Lysine	45	49.2 ± 4.1	109.2 ± 9.1
Methionine + Cysteine	22	27.0 ± 2.3	122.5 ± 10.6
Phenylalanine + Tyrosine	38	67.7 ± 6.6	178.0 ± 17.4
Threonine	23	31.7 ± 2.2	137.9 ± 9.6
Tryptophan	6	7.1 ± 0.6	119.0 ± 9.2
Valine	39	32.9 ± 3.8	84.3 ± 9.8

^1^ Values are means ± standard deviation (*n* = 3). * FAO [47].

**Table 8:** Nutritional contribution (%) of boiled common octopus in terms of mineral elements and EPA+DHA, taking into account a meal portion of 150 g.

	Adult	DRVs ^1^: AI or PRI (mg/day) ^2^	Nutritional Contribution (%)
Macroelements			
Mg	Men	350 ^3^	24.0 ± 1.0
	Women	300 ^3^	28.0 ± 1.2
P	Men/Women	550 ^4^	56.0 ± 3.6
K	Men/Women	3500 ^5^	10.4 ± 1.2
Na	Men/Women	2000 ^6^	24.9 ± 1.8
Microelements			
Cu	Men	1.6 ^7^	25.5 ± 5.3
	Women	1.3 ^7^	31. 3± 6.5
Zn	Men	9.4 ^8^	26.5 ± 1.4
	Women	7.5 ^8^	33.2 ± 1.7
Se	Men/Women	0.07 ^9^	75.6 ± 2.9
I	Men/Women	0.15 ^10^	12.4 ± 1.1
*n*-3 fatty acids			
EPA+DHA	Men/Women	250 ^11^	180.8 ± 9.4

Values are means ± standard deviation (*n* = 3). ^1^ The Dietary Reference Values (DRVs) are presented for adults; ^2^ Adequate Intakes (AIs) are presented in ordinary type and Population Reference Intakes (PRIs) in bold type; ^3^ EFSA [40]; ^4^ EFSA [41]; ^5^ EFSA [43]; ^6^ EFSA [44]; ^7^ EFSA [39]; ^8^ considering a phytate intake level of 300 mg/day [38]; ^9^ EFSA [37]; ^10^ EFSA [36]; ^11^ AI based on considerations of cardiovascular health [42].
